# Knee OA cost comparison for hyaluronic acid and knee arthroplasty

**DOI:** 10.1186/s13018-020-01848-7

**Published:** 2020-08-06

**Authors:** Kevin L. Ong, Faizan Niazi, Edmund Lau, Michael A. Mont, Andrew Concoff, Peter Shaw, Steven M. Kurtz

**Affiliations:** 1 Exponent, Inc., 3440 Market St, Suite 600, Philadelphia, PA 19104 USA; 2 Ferring Pharmaceuticals, Inc., Parsippany, NJ USA; 3 Exponent, Inc., Menlo Park, CA USA; 4 Lenox Hill Hospital, New York, NY USA; 5 United Rheumatology, Hauppauge, NY USA

**Keywords:** Hyaluronic acid, Direct costs, Knee arthroplasty, Economic burden

## Abstract

**Background:**

Limiting treatment to those recommended by the American Academy of Orthopaedic Surgeon Clinical Practice Guidelines has been suggested to decrease costs by 45% in the year prior to total knee arthroplasty, but this only focuses on expenditures leading up to, but not including, the surgery and not the entire episode of care. We evaluated the treatment costs following knee osteoarthritis (OA) diagnosis and determined whether these are different for patients who use intra-articular hyaluronic acid (HA) and/or knee arthroplasty.

**Methods:**

Claims data from a large commercial database containing de-identified data of more than 100 million patients with continuous coverage from 2012 to 2016 was used to evaluate the cumulative cost of care for over 2 million de-identified members with knee OA over a 4.5-year period between 2011 and 2015. Median cumulative costs were then stratified for patients with or without HA and/or knee arthroplasty.

**Results:**

Knee OA treatment costs for 1,567,024 patients over the 4.5-year period was $6.60 billion (mean $4210/patient) as calculated by the authors. HA and knee arthroplasty accounted for 3.0 and 61.5% of the overall costs, respectively. For patients who underwent knee arthroplasty, a spike in median costs occurred sooner for patients without HA use (around the 5- to 6-month time point) compared to patients treated with HA (around the 16- to 17-month time point).

**Conclusions:**

Non-arthroplasty therapies, as calculated by the authors, accounted for about one third of the costs in treating knee OA in our cohort. Although some have theorized that limiting the use of HA may reduce the costs of OA treatment, HA only comprised a small fraction (3%) of the overall costs. Among patients who underwent knee arthroplasty, those treated with HA experienced elevated costs from the surgery later than those without HA, which reflects their longer time to undergoing knee arthroplasty. The ability to delay or avoid knee arthroplasty altogether can have a substantial impact on the cost to the healthcare system.

## Introduction

Osteoarthritis (OA) is a degenerative disease that affects more than 30 million people in the USA [1]. In addition to pain and decreased quality of life, work productivity is also negatively affected by OA [2]. Indirect costs due to workplace absenteeism from osteoarthritis have been estimated to exceed $10 billion annually [2, 3]. More than 700,000 total knee arthroplasties (TKA) are performed annually in the USA to treat knee OA [4, 5]. With the increasing prevalence of knee OA, there are concerns that the number of TKAs will continue to increase over time [4]. Knee OA contributes to over $27 billion in annual healthcare costs, with the expenditures related to TKA exceeding $11 billion annually [1, 6, 7].

Knee OA is treated using a wide spectrum of therapies, including corticosteroid (CS) injections, physical therapy (PT) modalities, braces, opioids, tramadol medications, nonsteroidal anti-inflammatory drugs (NSAIDs), and hyaluronic acid (HA) injections [8]. The treatments recommended by the American Academy of Orthopaedic Surgeons (AAOS) in their Clinical Practice Guidelines (CPG) include PT, NSAIDs, and tramadol [9]. On the other hand, CS or HA injections, opioids, or knee braces were not recommended or had inconclusive evidence according to the AAOS CPG. It has been estimated that pre-surgical intervention costs could be reduced by up to 45% if only therapies recommended by the AAOS CPG were utilized by physicians [8]. However, previous cost studies primarily focus on the expenditures leading up to TKA, but not the entire episode of care (cost of TKA and post-operative care) [8, 10, 11]. Losina et al. found that TKA accounted for up to 61% of direct OA-related medical costs [12]. They also estimated that expanding eligibility criteria for TKA will lead to greater use of primary and revision TKAs and a 29% increase in lifetime knee OA-related healthcare costs. Moreover, primary TKAs have been estimated to increase substantially in the next two decades [4, 13, 14], which could substantially inflate total healthcare costs related to knee OA [4, 15]. Moreover, readmission following TKA [15] and the rising burden of revision TKA [4] will further fuel the healthcare costs.

With the rising costs from arthroplasty use, therapies that allow patients to avoid or delay surgery may help in reducing overall healthcare costs. For example, a number of professional societies [16–20], as well as the Department of Veteran Affairs and Department of Defense [21], have recommended the use of HA for only selective patients despite conflicting conclusions in the AAOS CPG [9]. Others have also strongly recommended HA for knee pain relief and potential disease-modifying effects, with greater evidence for patients with mild to moderate knee OA [22]. Moreover, previous studies have reported an association between HA use and longer time to knee arthroplasty [23, 24], which may help with delaying the costs attributed to the procedure. Furthermore, if knee arthroplasty can be delayed by a less costly alternative treatment, then it will be performed on older patients, which may help alleviate the increased risk of revision in younger patients [25].

This study aimed to address two research questions: (1) What is the contribution of HA and other therapies or resource utilization (e.g., physical therapy modalities, opioid prescriptions, primary knee arthroplasties) to the total cost of knee OA patient care? and (2) Does HA influence the timing of costs associated with patient care after knee OA diagnosis? We hypothesized that HA is a relatively minor (< 10%) contributor to the total costs of treating knee OA and that it would delay the costs of TKA. As previous studies have reported an association between HA use and longer time to knee arthroplasty [23, 24], this study sought to observe whether there are longer time to elevated costs, which would be indicative of the extended time to knee arthroplasty for HA patients.

## Methods

De-identified members who had knee OA were ascertained from a large commercial claims database (Health Intelligence Company LLC, Chicago, IL), containing Health Insurance Portability and Accountability Act (HIPAA) compliant de-identified data for more than 100 million patients with continuous coverage from January 1, 2011, to December 31, 2015. The dataset includes claims data representing every metropolitan statistical area in the USA. All medical/prescription claims and membership/provider data are collected in the dataset and represent total allowed amounts. Allowed amounts are the paid amount plus any member share, not including member premiums. This amount excludes any network discounts or noncovered amounts.

Patients with diagnosed knee OA were included in the study, as identified using the presence of principal or secondary International Classification of Diseases (ICD) diagnosis codes for knee OA (Additional file 1: Table 1). Patients diagnosed with nonspecific OA and knee pain on the same claim were also included. Patients were required to have no prior history of knee OA, based on a 6-month look-back period for no prior knee OA diagnosis. Due to the look-back criterion, knee OA patients diagnosed in the first 6 months of 2011 were excluded from the study because they did not have the requisite 6-month look-back period. Patients were excluded if they did not have a minimum 6 months continuous enrollment during the study period. A total of 43.67 million to 48.21 million members were enrolled annually during the period from 2011 to 2015, from which 1,567,024 knee OA patients were ascertained based on the inclusion and exclusion criteria. Population-level patient characteristics by sex, age, and census region were determined for all knee OA patients, as well as knee arthroplasty patients and HA patients (Table 1).

**Table 1:** Specific cost categories

Category	ICD procedure or HCPCS codes
HA	J7321, J7323 to J7327 (with diagnosis code of 711.x6, 712.x6, 715.x6, 716.x6, 717.x, 718.x6, 719.x6, 836.x, 844.x on the same claim as injection)
Primary knee arthroplasty	81.54, 0SRC0J* (without 0SPC* on the same claim), 0SRT0J* (without 0SPC0* on the same claim), 0SRU0J* (without 0SPD0* on the same claim), 0SRV0J* (without 0SPC0* on the same claim), 0SRW0J* (without 0SPD0* on the same claim), 0SRC0L*, 0SRD0J* (without 0SPD*on the same claim), 0SRD0L*, 27446, 27447
Revision knee arthroplasty	80.06, 81.55, 00.8x, 84.56, 84.57, 0SWC*JZ, 0SWD*JZ, (0SRC0JZ with 0SPC*), (0SRD0JZ with 0SPD*), (0SRV0JZ with 0SPC0*), (0SRW0JZ with 0SPD0*), (0SRT0JZ with 0SPC0*), (0SRU0JZ with 0SPD0*), (0SUW09Z with 0SPD0*), (0SUC0JZ with 0SPC0*), (0SUT09Z with 0SPC0*), (0SUD0JZ with 0SPD0*), (0SUU09Z with 0SPD0*), (0QRD0JZ with 0QPD0*), (0QUD0JZ with 0QPD0*), (0SUC09C with 0QPD0*), (0QRF0JZ with 0QPF0*), (0QUF0JZ with 0QPF0*), (0SUD09C with 0QPF0*), (0SUV09Z with 0SPC0*), (0SUW09Z with 0SPD0*), 0SHB08Z, 0SH908Z, 0SHC08Z, 0SHD08Z, 0SPC08Z, 0SPD08Z, 0SPB08Z, 0SP908Z, 27091, 27486, 27487, 11981, 11982
Physical therapy	97012, 97014, 97016, 97022, 97032, 97034, 97035, 97036, 97110, 97112, 97113, 97116, 97140, 97150, 97530, G0151
Intra-articular corticosteroid	J0702, J0704 (expired after Dec 31, 2010), J1020, J1030, J1040, J1094, J1100, J1700, J1710, J1720, J2650, J2920, J2930, J3300, J3301, J3302, J3303 (eff Jan 1, 2009)
Arthrocentesis	20610
Knee arthroscopy	80.26, 80.6, 80.76, 80.86, 81.45, 81.46, 81.47, 0SJC4ZZ, 0SJD4ZZ, 0SQC4ZZ, 0SQD4ZZ, 0SBD4ZZ, 0SBC4ZZ, 0S9C40Z, 0S9D40Z, 29866 to 29868, 29870 to 29871, 29873 to 29877, 29879 to 29888, 29999, G0289
Knee brace	L1800, L1810, L1812, L1820, L1830, L1831, L1832, L1833, L1834, L1836, L1840, L1843, L1844, L1845, L1846, L1847, L1848, L1850, L1860, L1870, L1880, K0901, K0902
Anesthesia for knee surgery	01400, 01402, 01404
Ultrasound/fluoroscopic imaging	20611 (eff 2015 onwards), 76942 or 77002
Knee imaging	73560, 73562, 73564, 73565, 73580, 73700 to 73702, 73706, 73721 to 73723, 73725, 76942

Knee OA-related claims, i.e., claims with a knee OA diagnosis, were compiled for the patients from the time of their knee OA diagnosis until the end of 2015. Cumulative costs (payments adjusted to November 2016 dollars) were determined on a monthly basis, using total allowed amounts, until the end of 2015 or the end of enrollment. The costs were stratified by patients who did and did not have a knee arthroplasty and by those who did and did not have HA use. The overall knee OA costs were determined, but the costs were also stratified into various specific categories (Additional file 1: Table 1): (1) HA, (2) primary knee arthroplasty, (3) revision knee arthroplasty, (4) physical therapy, (5) intra-articular corticosteroid, (6) arthrocentesis, (7) knee arthroscopy, (8) knee brace, (9) anesthesia for knee surgery, (10) ultrasound/fluoroscopic imaging, (11) knee imaging, (12) office visits, (13) pharmacy prescription claims, and (14) others (for remaining costs). Office visits were identified as those claims with the office as the site of service, but were not included in the above specific categories. Pharmacy prescriptions that were filled within 7 days of a knee OA-related claim were included in the analysis. HA was distinguished from pharmacy prescriptions because the HA usage was based on J codes, which include drugs and biologicals that are administered by the medical provider and are found in the professional claims, whereas pharmacy prescriptions are self-administered drugs that are found in the pharmacy claims.

In addition to the overall cost and the corresponding cost contributors, the median cost per patient was also evaluated for patients who did and did not have knee arthroplasty, as well as did and did not have HA use. The temporal trends in the per-patient median cost over time following knee diagnosis were compared. The median cumulative cost per patient after 1, 2, 3, and 4 years following knee OA diagnosis was also calculated. The 25th and 75th percentile costs per patient were also examined to provide an understanding of the distribution in costs.

## Results

**Fig. 1:**
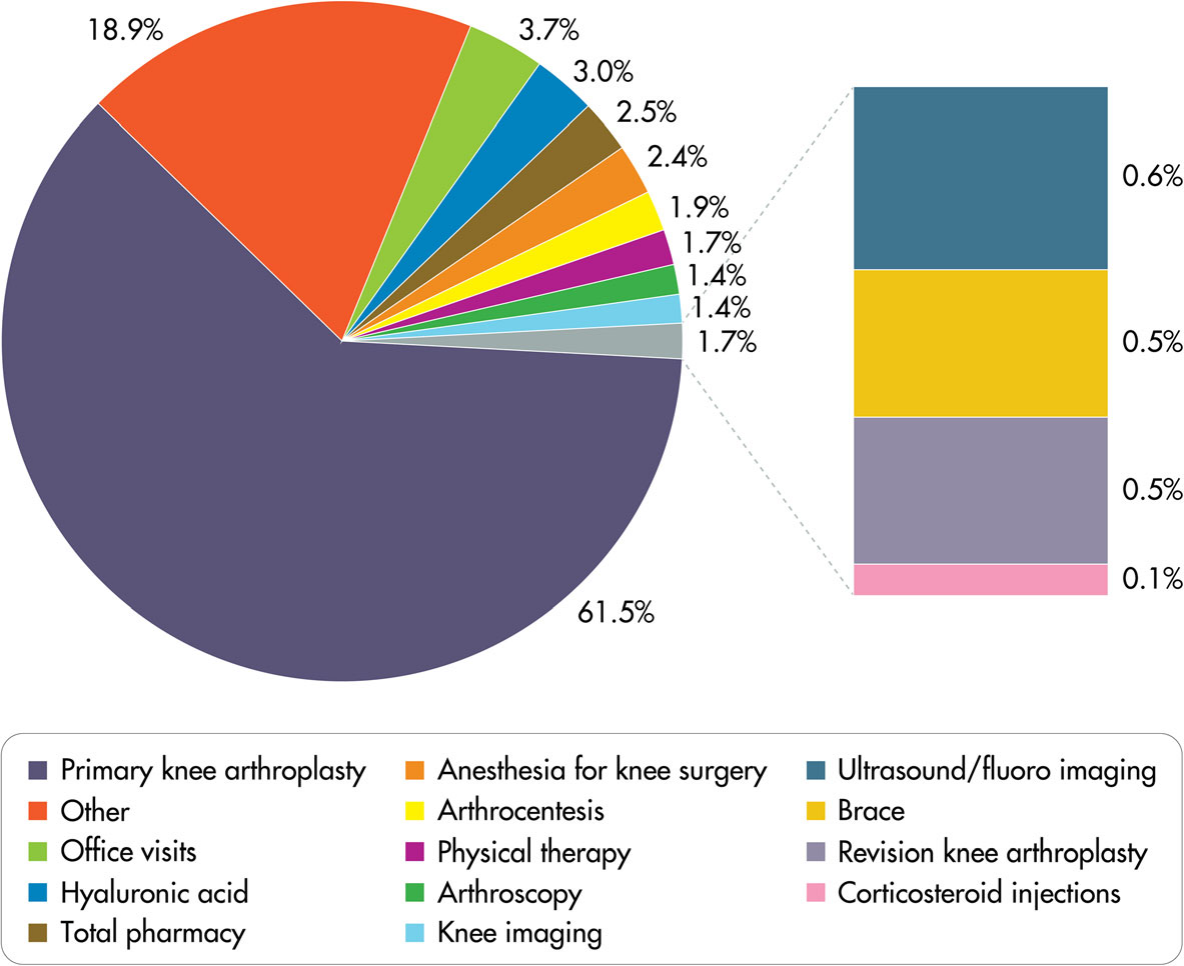
Contributions of various therapies to the overall cost of treating 1,567,024 knee OA patients. As calculated by the authors, the top five largest contributors to the non-specific “other” category included other/unspecified operations on skin/subcutaneous tissue (US$ 43.8 million), subsequent total hip arthroplasties (US$ 39.6 million), subsequent lumbar spine fusions (US$ 10.5 million), continuous passive motion devices (US$ 9.0 million), and unclassified drugs (US$ 4.7M)

The overall cost of treating the OA patients during the study period as calculated by the authors was US$ 6.60 billion (US$ 4210 per patient). Primary knee arthroplasty was the largest cost contributor with a conservative estimate of US$ 4.06 billion (61.5%) (Fig. 1), even though the majority of patients (91.2%) did not undergo a knee arthroplasty. Related arthroplasty costs (e.g., anesthesia, knee imaging) and post-arthroplasty costs (e.g., post-operative physical therapy, post-operative office visits, revision surgery, continuous passive motion devices) accounted for a further 11.6% of total costs. The mean primary knee arthroplasty cost was about US$ 29,300 per patient in our study population. The next largest specific cost categories were office visits, HA, prescription drugs, anesthesias for knee surgery, arthrocenteses, physical therapy visits, arthroscopies, knee imaging studies, knee braces, and corticosteroid injections. The majority of the prescription drugs were attributed to opioid prescriptions, accounting for 67% of the prescription pharmacy costs (Table 2). The top five largest contributors to the non-specific “other” category included other/unspecified operations on skin/subcutaneous tissue (US$ 43.8 million), subsequent total hip arthroplasties (US$ 39.6 million), subsequent lumbar spine fusions (US$ 10.5 million), continuous passive motion devices (US$ 9.0 million), and unclassified drugs (US$ 4.7 million).

**Table 2:** Contributions of various therapies (in US$) to the overall cost of treating knee OA patients with and without knee arthroplasty, as well as with and without HA use

	With knee arthroplasty		Without knee arthroplasty		With and without knee arthroplasty		
Categories	No HA	Any HA	No HA	Any HA	No HA	Any HA	All patients
Primary knee arthroplasty	$2,994,911,900	$1,061,612,700	$0	$0	$2,994,911,900	$1,061,612,700	$4,056,524,600
Revision knee arthroplasty	$24,414,900	$7,335,200	$0	$0	$24,414,900	$7,335,200	$31,750,100
Hyaluronic acid	$0	$33,368,300	$0	$165,567,400	$0	$198,935,700	$198,935,700
Corticosteroid	$677,900	$579,600	$3,990,200	$1,719,500	$4,668,100	$2,299,100	$6,967,200
Physical therapy	$44,093,300	$21,198,300	$33,304,200	$12,671,700	$77,397,500	$33,870,000	$111,267,500
Arthroscopy	$10,238,400	$5,083,500	$65,144,900	$13,142,200	$75,383,300	$18,225,700	$93,609,000
Brace	$3,873,500	$3,477,200	$14,580,800	$10,370,200	$18,454,300	$13,847,400	$32,301,700
Ultrasound/fluoro imaging	$2,044,600	$4,822,200	$7,258,900	$25,657,800	$9,303,500	$30,480,000	$39,783,500
Anesthesia for knee surgery	$105,795,100	$40,206,200	$10,679,800	$2,202,300	$116,474,900	$42,408,500	$158,883,400
Arthrocentesis	$7,495,300	$15,140,500	$41,151,100	$63,020,000	$48,646,400	$78,160,500	$126,806,900
Knee imaging	$13,289,600	$7,473,700	$53,768,700	$15,433,100	$67,058,300	$22,906,800	$89,965,100
Office visits	$33,027,900	$19,742,800	$138,959,900	$51,145,900	$171,987,800	$70,888,700	$242,876,500
Total pharmacy	$22,150,600	$17,666,500	$80,300,500	$44,926,000	$102,451,100	$62,592,500	$165,043,600
Opioids	$15,314,400	$11,537,300	$53,955,800	$29,549,400	$69,270,200	$41,086,700	$110,356,900
NSAIDs	$6,836,100	$6,125,500	$26,344,700	$15,376,800	$33,180,800	$21,502,300	$54,683,100
Others	$310,527,000	$122,053,300	$703,941,000	$108,963,900	$1,014,468,000	$231,017,200	$1,245,485,200
Total	$3,572,540,000	$1,359,760,000	$1,153,080,000	$514,820,000	$4,725,620,000	$1,874,580,000	$6,600,200,000

A total of 216,523 patients (13.8%) underwent HA therapy during the study period. The vast majority of the HA patients (83.5%) did not undergo primary knee arthroplasty during the study period. For the 16.5% of HA patients who subsequently underwent knee arthroplasty during the study period, HA contributed to 2.5% of their overall knee OA treatment costs compared to knee arthroplasty, which contributed 78.1% (Table 2). For the HA patients who did not undergo knee arthroplasty, HA was the largest cost contributor, accounting for 32.2% of the total cost. For the patients who did not use HA (“no HA” group), 7.6% (*n* = 102,812) underwent primary knee arthroplasty. Primary knee arthroplasty costs accounted for 83.8% of the overall costs for those no HA patients with primary knee arthroplasty.

**Fig. 2:**
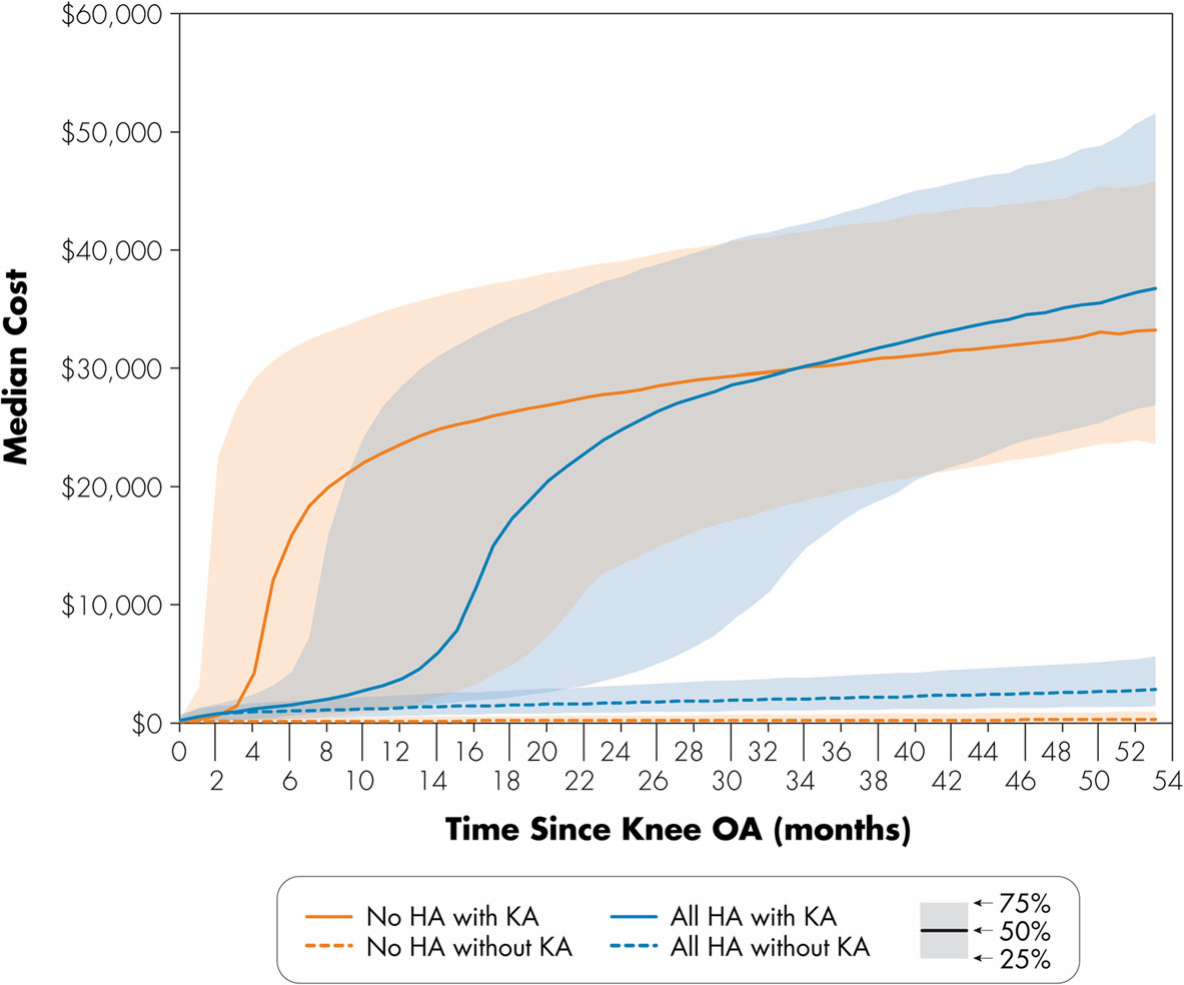
Median cost for HA and no-HA patients with and without knee arthroplasty in the months following knee OA diagnosis (with 25th and 75th percentile bands)

For patients who underwent knee arthroplasty within the 4.5 years following knee OA diagnosis, a spike in the median costs was observed, which reflects the cost of the knee arthroplasty (Fig. 2). This occurred sooner for patients who did not have HA use, with their inflection point around the 5- to 6-month time point compared to the 16- to 17-month time point for patients treated with HA. This temporal difference was also evident from the median costs at 1 year following knee diagnosis being greater for patients without HA who went on to knee arthroplasty compared to their HA counterparts (Table 3). Conversely, patients who did not undergo knee arthroplasty up to 4 years following knee OA diagnosis incurred lower costs than their knee arthroplasty counterparts.

**Table 3:** Median cumulative cost per patient after 1, 2, 3, and 4 years following knee OA diagnosis, with inter-quartile range in parenthesis (in US$)

Time points	No HA		All HA	
	Without KA, *n* = 1,247,689	With KA, *n* = 102,812	Without KA, *n* = 180,862	With KA, *n* = 35,661
**1 year**	$190 ($70–$528)	$22,952 ($1075–$34,872)	$1268 ($609–$2275)	$3230 ($1218–$26,847)
**2 years**	$219 ($80–$642)	$27,833 ($12,706–$38,932)	$1712 ($908–$3126)	$24,009 ($3597–$37,366)
**3 years**	$247 ($90–$762)	$30,271 ($19,232–$41,922)	$2110 ($1123–$3963)	$30,607 ($16,006–$42,745)
**4 years**	$289 ($106–$901)	$32,320 ($22,707–$44,261)	$2562 ($1346–$4915)	$34,803 ($24,286–$47,508)

## Discussion

In our study of knee OA patients with commercial coverage, non-arthroplasty therapies accounted for about one third of the costs in treating knee OA in our cohort of younger patients, with HA comprising a small fraction of the costs. Moreover, adding related arthroplasty costs and post-arthroplasty costs would further decrease the non-arthroplasty cost contributions to about a quarter of the overall costs. Of the HA patients, 16.5% subsequently underwent knee arthroplasty during the study period, but HA contributed to 2.5% of their overall knee OA treatment costs compared to knee arthroplasty, which contributed 78.1%. Among patients who went on to knee arthroplasty, those treated with HA experienced elevated costs from the surgery later than those without HA, which reflects the longer time to knee arthroplasty for the HA patients. Regardless of whether the patients used HA or not, those who had a knee arthroplasty had greater median costs of about US$ 32,000 at 4 years post-knee OA diagnosis.

This study has several limitations. The current results were based on privately insured patients from large commercial health plans and may not reflect the experience of all privately insured or Medicare knee OA patients. Since the patient population was focused on younger patients, it may have included those with post-traumatic, genetic, and other premature forms of OA that may not mirror the natural history of the typical older OA patient. However, the present study with more than 1.5 million patients provides the real-world economic burden of treating knee OA for a major insurer in the USA: the current study cohort is estimated to represent about 10% of the knee OA patients in the USA [26]. The economic burden was also derived from a direct cost payer perspective, which underestimated the true costs because it does not include any indirect costs. The indirect costs have been shown to potentially exceed the direct costs in some healthcare systems [27]. Due to the use of administrative claims data, the severity of knee OA could not be examined for the patients. Those who had milder severity may be less appropriate candidates for knee arthroplasty. The relationship between comorbidities and costs was not within the scope of the present study. It was also unclear if the majority of patients who did not undergo knee arthroplasty were either not candidates for knee arthroplasty or able to avoid arthroplasty due to relief from the use of other therapies.

The economic burden of treating knee OA varies depending on the patient population and healthcare system. The present study of the direct costs would extrapolate to about US$ 14 billion in annual costs for an estimated 15.17 million symptomatic knee OA patients in the USA [26]. Comparatively, Bertin et al. reported that the annual healthcare cost per knee OA patient in France was approximately € 454 in 2010 [28] (approximately US$ 670 in 2016). On the other hand, Leardini and coworkers estimated that the direct economic burden for knee OA in Italy was € 934 per patient annually (approximately US$ 1140 in 2016) [29]. Osteoarthritis patients in Australia incurred a mean of AU$ 127 to AU$ 651 (approximately US$ 160 to US$ 820 in 2016), depending on their age and sex [30]. The mean direct cost of osteoarthritis in Canada has been estimated to be Can$ 811 per year (approximately US$ 900 in 2016), with the costs increasing by sevenfold if the patient had undergone primary joint arthroplasty compared to those who had not seen an orthopedic surgeon [31]. Moreover, although the vast majority of patients in the present study did not undergo knee arthroplasty, this procedure was still the largest cost contributor to treating knee OA. The US$ 4.06 billion (61.5%) that was attributed to knee arthroplasty was a conservative amount because it did not include any related intervention costs (e.g., anesthesia, knee imaging), nor post-arthroplasty costs (e.g., physical therapy, office visits, revision surgery), which would have added at least another 11.6% of the overall costs. HA, along with knee arthroscopies, knee braces, and corticosteroids, are therapies that the AAOS CPG indicated had inconclusive evidence or were not recommended [9]. However, based on the present estimates, these therapies each contributed 3% or less to the overall knee OA-related direct costs.

For HA patients who subsequently underwent knee arthroplasty, HA only contributed 2.5% to the total cost in the present study. This contrasts with others who reported that about 25 to 29% of costs are due to HA [8, 11], but those estimates were limited by their evaluation of costs 12 months prior to knee arthroplasty and, more importantly, failure to consider inpatient costs and surgical costs, including the knee arthroplasty costs. On the other hand, Cohen et al. [10] included inpatient and procedural costs in their evaluation of the charges within the 2-year period before total knee arthroplasty for Medicare and privately insured patients, but they did not consider the knee arthroplasty costs. When the surgical costs were included in the Cohen study, injections only contributed about 3% of the overall costs, a similar finding to our study. Also, similar to our study, the primary cost contributors to the direct costs of OA in Australia were also hospital inpatient costs (43%), followed by residential care and/or rehabilitation costs (32%) and pharmaceutical costs (11%) [32]. Berger and coworkers [33] also reported that the mean healthcare costs per patient in the 2 years prior to, but not including, knee arthroplasty was US$ 20,464, of which outpatient care, pharmacotherapy, and inpatient care represented 45%, 21%, and 19%, respectively. HA was found to only represent 1.2% of the mean costs, similar to our study. Costs following knee OA was also examined in a recent study [34], but only evaluated the costs over a 2-year period compared to 4 years in the present study. Moreover, the authors did not evaluate temporal changes in per-patient cost following knee OA and the corresponding extended time to cost increases for HA and knee arthroplasty patients.

Among patients who went on to knee arthroplasty, those who had HA experienced elevated costs from the surgery by nearly a year later than those who did not have HA, which reflects the longer time to knee arthroplasty for the HA patients. While previous studies have reported the association between HA use and longer time to knee arthroplasty [23, 24], cost was not examined. In a study of primarily privately insured and self-insured patients, Altman et al. [23] reported longer median time to TKA of about 12 months for patients who received HA versus those who did not. Longer median time to knee arthroplasty of about 9 months was also observed for patients who received HA in a study of elderly Medicare knee OA population by Ong et al. [24]. Potential implications of an extension to the time to knee arthroplasty include one-time cost savings to yield a lower present discounted cost of surgery or patient attrition before the surgery or subsequent revision [35], as well as an opportunity for patients to better control their comorbidities prior to surgery, which in turn could help with reducing post-operative morbidity [36, 37]. HA has been described to act through various mechanisms, such as joint lubrication and anti-inflammatory effects, but may depend on product-specific molecular weight [38]. The role of corticosteroids in extending time to knee arthroplasty in the present study was not investigated, but others have found that patients who received intra-articular corticosteroids were associated with an extended time to surgery, which was further extended when paired with HA, suggesting a potential synergistic effect [24].

## Conclusion

Non-arthroplasty therapies accounted for about one third of the costs (38.5%) in treating knee OA in our cohort of younger patients, with HA comprising a small fraction of the costs (3.0%). Most HA patients avoided primary knee arthroplasty during the study period. Other non-arthroplasty therapies included prescription drugs (2.5% of total costs), arthrocentesis (1.9%), physical therapy (1.7%), and arthroscopy (1.4%). With the wide spectrum of therapies to treat knee OA, efforts to identify the most appropriate candidates for arthroplasty and non-arthroplasty therapies can help reduce costs to the healthcare system. For appropriate patients, the ability to delay or avoid knee arthroplasty altogether using a less expensive, alternative therapy can have a substantial impact on the cost to the healthcare system.

## Supplementary information

**Additional file 1: Table 1.** ICD-9 and ICD-10 codes for knee OA.

## Data Availability

All data generated or analyzed during this study are included in this published article.
