# Supplementary material for: Knee OA cost comparison for hyaluronic acid and knee arthroplasty
Source: J Orthop Surg Res. 2020 Aug 6;15:305. doi: 10.1186/s13018-020-01848-7 (PMC7412646; doi:10.1186/s13018-020-01848-7)
Supplement: Supplementary file 1 — Additional file 1: Table 1. ICD-9 and ICD-10 codes for knee OA. [file 13018_2020_1848_MOESM1_ESM.docx]

Appendix Table 1. ICD-9 and ICD-10 codes for knee OA

| *Diagnosis* | *ICD-9 diagnosis code* | *ICD-10 diagnosis code* |
| --- | --- | --- |
| Knee OA | 715.06, 715.16, 715.26, 715.36, 715.86, or 715.96 | M17.0, M17.1, M17.10, M17.11, M17.12, M17.2, M17.3, M17.30, M17.31, M17.32, M17.4, M17.5, or M17.9 |
| Non-specific OA and knee pain | (715.08, 715.18, 715.28, 715.38, 715.88, or 715.98) and 719.46 on the same claim | (M19.90, M19.91, or M19.93) and (M25.561, M25.562, or M25.569) on the same claim |
|  | (715.09, 715.19, 715.29, 715.39, 715.89, or 715.99) and 719.46 on the same claim | (M15.0, M15.3, or M15.8), and (M25.561, M25.562, or M25.569) on the same claim |
|  | (715.00, 715.10, 715.20, 715.30, 715.80, or 715.90) and 719.46 on the same claim | (M15.0, M15.4, M15.8, M15.9, M19.90, M19.91, M19.92, or M19.93)  and (M25.561, M25.562, or M25.569) on the same claim |
